# Anisotropic Picone identities and anisotropic Hardy inequalities

**DOI:** 10.1186/s13660-017-1292-4

**Published:** 2017-01-13

**Authors:** Tingfu Feng, Xuewei Cui

**Affiliations:** Department of Applied Mathematics, Northwestern Polytechnical University, Xi’an, Shaanxi 710072 P.R. China

**Keywords:** 26D10, 26D15, anisotropic Picone identity, anisotropic Hardy type inequality, anisotropic elliptic equation, Sturmian comparison principle

## Abstract

In this paper, we derive an anisotropic Picone identity for the anisotropic Laplacian, which contains some known Picone identities. As applications, a Sturmian comparison principle to the anisotropic elliptic equation and an anisotropic Hardy type inequality are shown.

## Introduction and main results

In recent years, the anisotropic Laplacian

1.1 $$ \sum_{i = 1}^{n} { \frac{\partial}{ {\partial{x_{i}}}} \biggl( {{{\biggl\vert {\frac{{\partial u}}{ {\partial{x_{i}}}}} \biggr\vert }^{{p_{i}} - 2}}\frac{{\partial u}}{ {\partial{x_{i}}}}} \biggr)} ,\quad {p_{i}} > 1, $$

has been considerably concerned. Note that if ${p_{i}} = 2$ (${i = 1, \ldots,n}$), then (1.1) becomes the classical Laplacian; if ${p_{i}} = p = \mathrm{const}$, then (1.1) is the pseudo-*p*-Laplacian (see [1])

$$\sum_{i = 1}^{n} {\frac{\partial}{ {\partial{x_{i}}}} \biggl( {{{\biggl\vert {\frac{{\partial u}}{ {\partial{x_{i}}}}} \biggr\vert }^{p - 2}} \frac{{\partial u}}{ {\partial{x_{i}}}}} \biggr)}. $$

The anisotropic Laplacian has not only the widespread practical background in the natural science, but also the important theoretical value in the mathematics. For example, it reflects anisotropic physical properties of some reinforced materials (Lions [2] and Tang [3]), and describes the dynamics of fluids in the anisotropic media when the conductivities of the media are different in each direction [4, 5]. The equations associated with (1.1) are also deduced in the image processing [6]. Existence, integrability, boundedness, and continuity of solutions to anisotropic elliptic equations have received much attention; see [7–15] and the references therein. In this paper, we prove an anisotropic Picone identity for the anisotropic Laplacian, which contains some known Picone identities. As applications, a Sturmian comparison principle to the anisotropic elliptic equation and an anisotropic Hardy type inequality are given. Before giving the main results of this paper, we briefly recall the existing results for the isotropic case.

Picone [16] considered the homogeneous linear second order differential system

$$\left \{ \textstyle\begin{array}{l} {{ ( {{a_{1}}(x)u'} )}^{\prime}} + {b_{1}}(x)u = 0, \\ {{ ( {{a_{2}}(x)v'} )}^{\prime}} + {b_{2}}(x)v = 0, \end{array}\displaystyle \right . $$

where *u* and *v* are differentiable functions in *x*, and proved the identity that, for the differentiable function $v(x) \ne0$,

1.2 $$ { \biggl( {\frac{u}{ v} \bigl( {{a_{1}}u'v - {a_{2}}uv'} \bigr)} \biggr)^{\prime}} = ( {{b_{2}} - {b_{1}}} ){u^{2}} + ( {{a_{1}} - {a_{2}}} ){u^{\prime 2}} + {a_{2}} { \biggl( {u' - v'\frac{u}{ v}} \biggr)^{2}}; $$

then a Sturmian comparison principle and the oscillation theory of solutions were obtained via (1.2). Picone [17] (see also Allegretto [18]) generalized (1.2) to a Laplacian that, for differentiable functions $v > 0$ and $u \geq0$,

1.3 $$\begin{aligned} { \biggl( {\nabla u - \frac{u}{ v}\nabla v} \biggr)^{2}} =& {\vert {\nabla u} \vert ^{2}} + \frac{{{u^{2}}}}{ {{v^{2}}}}{\vert {\nabla v} \vert ^{2}} - 2\frac{u}{ v} \nabla v \cdot\nabla u \\ =& {\vert {\nabla u} \vert ^{2}} - \nabla \biggl( {\frac{{{u^{2}}}}{ v}} \biggr)\nabla v. \end{aligned}$$

Allegretto and Huang [19], Dunninger [20] independently extended (1.3) to a *p*-Laplacian, for differentiable functions $v > 0$ and $u \geq0$,

1.4 $$\begin{aligned}& {\vert {\nabla u} \vert ^{p}} + (p - 1) \frac{{{u^{p}}}}{ {{v^{p}}}}{\vert {\nabla v} \vert ^{p}} - p\frac{{{u^{p - 1}}}}{ {{v^{p - 1}}}}{ \vert {\nabla v} \vert ^{p - 2}}\nabla v \cdot\nabla u \\& \quad = {\vert {\nabla u} \vert ^{p}} - \nabla \biggl( { \frac{{{u^{p}}}}{ {{v^{p - 1}}}}} \biggr){\vert {\nabla v} \vert ^{p - 2}}\nabla v, \end{aligned}$$

and applied (1.4) to derive a Sturmian comparison principle, Liouville’s theorem, the Hardy inequality, and some profound results for *p*-Laplace equations and systems. For other generalizations of the Picone identities and applications, see Bal [21], Dwivedi [22], Dwivedi and Tyagi [23], Niu, Zhang and Wang [24], Tyagi [25]. These results indicate that Picone identities are seemingly simple in form, but extremely useful in the study of partial differential equations, and they have become an important tool in the analysis.

Our main results are as follows.

### Theorem 1.1

Anisotropic Picone identity

*Let* $v > 0$ *and* $u \geq0$ *be two differentiable functions in the set* $\Omega \subset{R^{n}}$, *and denote*

1.5 $$\begin{aligned}& R(u,v) = \sum_{i = 1}^{n} {{{ \biggl\vert {\frac{{\partial u}}{ {\partial{x_{i}}}}} \biggr\vert }^{{p_{i}}}}} - \sum _{i = 1}^{n} {\frac {\partial}{ {\partial{x_{i}}}} \biggl( { \frac{{{u^{{p_{i}}}}}}{ {{v^{{p_{i}} - 1}}}}} \biggr){{\biggl\vert {\frac{{\partial v}}{ {\partial{x_{i}}}}} \biggr\vert }^{{p_{i}} - 2}}\frac{{\partial v}}{ {\partial{x_{i}}}}}, \end{aligned}$$

1.6 $$\begin{aligned}& L(u,v)= \sum_{i = 1}^{n} {{{ \biggl\vert {\frac{{\partial u}}{ {\partial{x_{i}}}}} \biggr\vert }^{{p_{i}}}}} - \sum _{i = 1}^{n} {{p_{i}}\frac {{{u^{{p_{i}} - 1}}}}{ {{v^{{p_{i}} - 1}}}}{{ \biggl\vert {\frac{{\partial v}}{ {\partial{x_{i}}}}} \biggr\vert }^{{p_{i}} - 2}} \frac{{\partial v}}{ {\partial{x_{i}}}}\frac{{\partial u}}{ {\partial{x_{i}}}}} \\& \hphantom{L(u,v)={}}{} + \sum_{i = 1}^{n} { ( {{p_{i}} - 1} )\frac{{{u^{{p_{i}}}}}}{ {{v^{{p_{i}}}}}}{{\biggl\vert {\frac{{\partial v}}{ {\partial{x_{i}}}}} \biggr\vert }^{{p_{i}}}}}, \end{aligned}$$

*where* ${p_{i}} > 1$ (${i = 1, \ldots,n}$). *Then*

1.7 $$ R(u,v) = L(u,v). $$

*Moreover*, *we have*

$$L(u,v) \geq0; $$

*furthermore*, $L(u,v) = 0$ *a*.*e*. *in* Ω *if and only if* $u = cv$ *a*.*e*. *in* Ω, *c* *is a positive constant*.

### Remark 1.2

If ${p_{i}} = 2$ (${i = 1, \ldots,n}$) in (1.5) and (1.6), we have (1.3) from (1.7). If ${p_{i}} = p = \mathrm{const}$ (${i = 1, \ldots,n}$) in (1.5) and (1.6), the result in [26] follows. Moreover, the identity in Theorem 1.1 is different from the one in [26].

### Theorem 1.3

Anisotropic Hardy type inequality

*Let* $u \in C_{0}^{1} ( A )$, $1 < {p_{i}} < n$, $i = 1, \ldots,n$, $A = \{ x \in{R^{n}}| {{x_{i}} \ne0,i = 1, \ldots,n} \}$. *Then we have*

1.8 $$ \sum_{i = 1}^{n} \int_{A} {\biggl\vert \frac{\partial u}{\partial x_{i}} \biggr\vert }^{p_{i}}\,dx \geq\sum_{i = 1}^{n} \biggl( \frac{p_{i} - 1}{p_{i}} \biggr)^{p_{i}} \int_{A} {\frac{{{{\vert u \vert }^{{p_{i}}}}}}{ {{{\vert {{x_{i}}} \vert }^{{p_{i}}}}}}}. $$

This paper is organized as follows: The proofs of Theorem 1.1 and a Sturmian comparison principle to the anisotropic elliptic equation are given in Section 2; Section 3 is devoted to the proof of Theorem 1.3 in which a key ingredient is to choose a suitable auxiliary function (see (3.3) below) for the anisotropic case. Two corollaries are also furnished.

## Proof of Theorem 1.1

### Proof of Theorem 1.1

One derives easily that

$$\begin{aligned} R(u,v) &= \sum_{i = 1}^{n} {{{\biggl\vert { \frac{{\partial u}}{ {\partial{x_{i}}}}} \biggr\vert }^{{p_{i}}}}} - \sum _{i = 1}^{n} {\frac {\partial}{ {\partial{x_{i}}}} \biggl( { \frac{{{u^{{p_{i}}}}}}{ {{v^{{p_{i}} - 1}}}}} \biggr){{\biggl\vert {\frac{{\partial v}}{ {\partial{x_{i}}}}} \biggr\vert }^{{p_{i}} - 2}}\frac{{\partial v}}{ {\partial{x_{i}}}}} \\ &= \sum_{i = 1}^{n} {{{\biggl\vert { \frac{{\partial u}}{ {\partial{x_{i}}}}} \biggr\vert }^{{p_{i}}}}} - \sum _{i = 1}^{n} {\frac {{{p_{i}}{u^{{p_{i}} - 1}}\frac{{\partial u}}{{\partial{x_{i}}}}{v^{{p_{i}} - 1}} - {u^{{p_{i}}}}({p_{i}} - 1){v^{{p_{i}} - 2}}\frac{{\partial v}}{{\partial{x_{i}}}}}}{ {{{ [ {{v^{{p_{i}} - 1}}} ]}^{2}}}}{{\biggl\vert { \frac{{\partial v}}{ {\partial{x_{i}}}}} \biggr\vert }^{{p_{i}} - 2}}\frac{{\partial v}}{ {\partial{x_{i}}}}} \\ &=\sum_{i = 1}^{n} {{{\biggl\vert { \frac{{\partial u}}{ {\partial{x_{i}}}}} \biggr\vert }^{{p_{i}}}}} - \sum _{i = 1}^{n} {{p_{i}}\frac {{{u^{{p_{i}} - 1}}}}{ {{v^{{p_{i}} - 1}}}}{{ \biggl\vert {\frac{{\partial v}}{ {\partial{x_{i}}}}} \biggr\vert }^{{p_{i}} - 2}} \frac{{\partial v}}{ {\partial{x_{i}}}}\frac{{\partial u}}{ {\partial{x_{i}}}}} + \sum_{i = 1}^{n} { ( {{p_{i}} - 1} )\frac {{{u^{{p_{i}}}}}}{ {{v^{{p_{i}}}}}}{{\biggl\vert { \frac{{\partial v}}{ {\partial{x_{i}}}}} \biggr\vert }^{{p_{i}}}}} \\ &=L(u,v), \end{aligned}$$

which is (1.7). To check $L(u,v) \geq0$, we rewrite $L(u,v)$ by

2.1 $$\begin{aligned} L(u,v) =&\sum_{i = 1}^{n} {{{ \biggl\vert {\frac{{\partial u}}{ {\partial{x_{i}}}}} \biggr\vert }^{{p_{i}}}}} - \sum _{i = 1}^{n} {{p_{i}}\frac {{{u^{{p_{i}} - 1}}}}{ {{v^{{p_{i}} - 1}}}}{{ \biggl\vert {\frac{{\partial v}}{ {\partial{x_{i}}}}} \biggr\vert }^{{p_{i}} - 1}}\biggl\vert { \frac{{\partial u}}{ {\partial{x_{i}}}}} \biggr\vert } + \sum_{i = 1}^{n} { ( {{p_{i}} - 1} )\frac{{{u^{{p_{i}}}}}}{ {{v^{{p_{i}}}}}}{{\biggl\vert { \frac{{\partial v}}{ {\partial{x_{i}}}}} \biggr\vert }^{{p_{i}}}}} \\ &{} + \sum_{i = 1}^{n} {{p_{i}} \frac{{{u^{{p_{i}} - 1}}}}{ {{v^{{p_{i}} - 1}}}}{{\biggl\vert {\frac{{\partial v}}{ {\partial{x_{i}}}}} \biggr\vert }^{{p_{i}} - 2}} \biggl\{ {\biggl\vert {\frac {{\partial v}}{ {\partial{x_{i}}}}} \biggr\vert \biggl\vert {\frac{{\partial u}}{ {\partial{x_{i}}}}} \biggr\vert - \frac{{\partial v}}{ {\partial{x_{i}}}} \frac{{\partial u}}{ {\partial{x_{i}}}}} \biggr\} } \\ :=& \mathit{I} + \mathit{II}, \end{aligned}$$

where

$$\begin{aligned}& \mathit{I} = \sum_{i = 1}^{n} {{p_{i}} \biggl[ {\frac{1}{ {{p_{i}}}}{{\biggl\vert { \frac{{\partial u}}{ {\partial{x_{i}}}}} \biggr\vert }^{{p_{i}}}} + \frac{{{p_{i}} - 1}}{ {{p_{i}}}}{{ \biggl( {{{ \biggl( {\frac{u}{ v}\biggl\vert {\frac{{\partial v}}{ {\partial{x_{i}}}}} \biggr\vert } \biggr)}^{{p_{i}} - 1}}} \biggr)}^{\frac{{{p_{i}}}}{{{p_{i}} - 1}}}}} \biggr]} \\& \hphantom{\mathit{I} ={}}{} - \sum_{i = 1}^{n} {{p_{i}}\frac{{{u^{{p_{i}} - 1}}}}{ {{v^{{p_{i}} - 1}}}}{{\biggl\vert {\frac{{\partial v}}{ {\partial{x_{i}}}}} \biggr\vert }^{{p_{i}} - 1}}\biggl\vert {\frac{{\partial u}}{ {\partial{x_{i}}}}} \biggr\vert }, \\& \mathit{II} = \sum_{i = 1}^{n} {{p_{i}}\frac{{{u^{{p_{i}} - 1}}}}{ {{v^{{p_{i}} - 1}}}}{{\biggl\vert {\frac{{\partial v}}{ {\partial{x_{i}}}}} \biggr\vert }^{{p_{i}} - 2}} \biggl\{ {\biggl\vert {\frac {{\partial v}}{ {\partial{x_{i}}}}} \biggr\vert \biggl\vert {\frac{{\partial u}}{ {\partial{x_{i}}}}} \biggr\vert - \frac{{\partial v}}{ {\partial{x_{i}}}} \frac{{\partial u}}{ {\partial{x_{i}}}}} \biggr\} }. \end{aligned}$$

Recall Young’s inequality: for $a \geq0$ and $b \geq0$,

2.2 $$ ab \leq\frac{{{a^{{p_{i}}}}}}{ p} + \frac{{{b^{{q_{i}}}}}}{ q}, $$

where ${p_{i}} > 1$, ${q_{i}} > 1$ ($i = 1, \ldots,n$) and $\frac{1}{ {{p_{i}}}} + \frac{1}{{{q_{i}}}} = 1$; the equality holds if and only if ${a^{{p_{i}}}} = {b^{{q_{i}}}}$, namely, $a = {b^{\frac{1}{{{p_{i}} - 1}}}}$. We take $a = \vert {\frac{{\partial u}}{{\partial {x_{i}}}}} \vert $ and $b = { ( {\frac{u}{v}\vert {\frac{{\partial v}}{{\partial{x_{i}}}}} \vert } )^{{p_{i}} - 1}}$ in (2.2) to obtain

2.3 $$\begin{aligned}& {p_{i}}\biggl\vert {\frac{{\partial u}}{ {\partial{x_{i}}}}} \biggr\vert { \biggl( {\frac{u}{ v}\biggl\vert {\frac{{\partial v}}{ {\partial{x_{i}}}}} \biggr\vert } \biggr)^{{p_{i}} - 1}} \\& \quad \leq{p_{i}} \biggl[ {\frac{1}{ {{p_{i}}}}{{\biggl\vert { \frac{{\partial u}}{ {\partial{x_{i}}}}} \biggr\vert }^{{p_{i}}}} + \frac{{{p_{i}} - 1}}{ {{p_{i}}}}{{ \biggl( {{{ \biggl( {\frac{u}{ v}\biggl\vert {\frac{{\partial v}}{ {\partial{x_{i}}}}} \biggr\vert } \biggr)}^{{p_{i}} - 1}}} \biggr)}^{\frac{{{p_{i}}}}{{{p_{i}} - 1}}}}} \biggr], \end{aligned}$$

and so $\mathit{I} \geq0$ from (2.3). Clearly, $\mathit{II} \geq0$ in virtue of $\vert {\frac{{\partial v}}{ {\partial{x_{i}}}}} \vert \vert {\frac{{\partial u}}{ {\partial{x_{i}}}}} \vert - \frac{{\partial v}}{ {\partial{x_{i}}}}\frac{{\partial u}}{ {\partial{x_{i}}}} \geq0$. Hence $L(u,v) \geq0$ from (2.1).

If $u = cv$, *c* is a positive constant, then clearly $L(u,v) = 0$. Now let us conclude that $L(u,v) = 0$ implies $u = cv$. In fact, if $L(u,v)({x_{0}}) = 0$, ${x_{0}} \in\Omega$, then we consider the two cases $u({x_{0}}) \ne0$ and $u({x_{0}}) = 0$, respectively.

(a) If $u({x_{0}}) \ne0$, then $\mathit{I} = 0$ and $\mathit{II} = 0$. One shows by $\mathit{I} = 0$ that

2.4 $$ \biggl\vert {\frac{{\partial u}}{ {\partial{x_{i}}}}} \biggr\vert = \frac{u}{ v}\biggl\vert {\frac{{\partial v}}{ {\partial{x_{i}}}}} \biggr\vert . $$

Using $\mathit{II} = 0$, it implies

2.5 $$ \frac{{\partial u}}{ {\partial{x_{i}}}} = c\frac{{\partial v}}{ {\partial{x_{i}}}}. $$

Putting (2.5) into (2.4) yields $u = cv$.

(b) If $u({x_{0}}) = 0$, then we denote $S = \{x \in\Omega | {u(x) = 0} \}$ and $\frac{{\partial u}}{{\partial {x_{i}}}} = 0$ a.e. in *S*. Thus

$$\frac{\partial}{ {\partial{x_{i}}}} \biggl( {\frac{u}{ v}} \biggr) = \frac{{v\frac{{\partial u}}{{\partial{x_{i}}}} - u\frac{{\partial v}}{{\partial{x_{i}}}}}}{ {{v^{2}}}} = 0, $$

which shows $u = cv$. The proof of Theorem 1.1 is completed. □

Let us address anisotropic Sobolev spaces; see Adams [27], Lu [28], Troisi [29] *etc.* Given a domain $\Omega \subset {R^{n}}$, ${p_{i}} > 1$, $i = 1,2, \ldots,n$. We define two anisotropic Sobolev spaces by

$${W^{1, ( {{p_{i}}} )}}(\Omega) = \biggl\{ {u \in {W^{1,1}}(\Omega): \frac{{\partial u}}{ {\partial{x_{i}}}} \in{L^{{p_{i}}}}(\Omega),i = 1, \ldots,n} \biggr\} $$

and

$$W_{0}^{1, ( {{p_{i}}} )}(\Omega) = \biggl\{ {u \in W_{0}^{1,1}( \Omega):\frac{{\partial u}}{ {\partial{x_{i}}}} \in{L^{{p_{i}}}}(\Omega),i = 1, \ldots,n} \biggr\} , $$

with the norms

$${\Vert u \Vert _{{W^{1, ( {{p_{i}}} )}}(\Omega)}} = \int _{\Omega}{ \vert u \vert \,dx} + \sum _{i = 1}^{n} {{{ \biggl( { \int_{\Omega}{{{\biggl\vert {\frac{{\partial u}}{ {\partial{x_{i}}}}} \biggr\vert }^{{p_{i}}}}\,dx} } \biggr)}^{\frac{1}{{{p_{i}}}}}}} $$

and

$${\Vert u \Vert _{W_{0}^{1, ( {{p_{i}}} )}(\Omega)}} = \sum_{i = 1}^{n} {{{ \biggl( { \int_{\Omega}{{{\biggl\vert {\frac{{\partial u}}{ {\partial{x_{i}}}}} \biggr\vert }^{{p_{i}}}}\,dx} } \biggr)}^{\frac{1}{{{p_{i}}}}}}} , $$

respectively. Note that $W_{0}^{1, ( {{p_{i}}} )}(\Omega)$ is the closure of $C_{0}^{\infty}(\Omega)$ in ${W^{1, ( {{p_{i}}} )}}(\Omega)$. It is well known that ${W^{1, ( {{p_{i}}} )}}(\Omega)$ and $W_{0}^{1, ( {{p_{i}}} )}(\Omega)$ are both separable and reflexive Banach spaces.

We will show a Sturmian comparison principle to the anisotropic elliptic equation by Theorem 1.1.

### Proposition 2.1

*Let* ${f_{1}}(x)$ *and* ${f_{2}}(x)$ *be two continuous functions with* ${f_{1}}(x) < {f_{2}}(x)$ *in the bounded domain* Ω. *Assume that there exists a positive function* $u \in W_{0}^{1, ( {{p_{i}}} )}(\Omega)$ *satisfying*

2.6 $$ \left \{ { \textstyle\begin{array}{l@{\quad}l} - \sum_{i = 1}^{n} {\frac{\partial}{{\partial{x_{i}}}} ( {{{\vert {\frac{{\partial u}}{{\partial{x_{i}}}}} \vert }^{{p_{i}} - 2}}\frac{{\partial u}}{{\partial{x_{i}}}}} ) = \sum_{i = 1}^{n} {{f_{1}}(x)} {u^{{p_{i}} - 1}}} , & x \in\Omega, \\ u > 0, & x \in\Omega, \\ u = 0, & x \in\partial\Omega. \end{array}\displaystyle } \right . $$

*Then any nontrivial solution* *v* *to the following anisotropic elliptic equation*:

2.7 $$ - \sum_{i = 1}^{n} { \frac{{{u^{{p_{i}}}}}}{ {{v^{{p_{i}} - 1}}}}\frac{\partial}{ {\partial{x_{i}}}} \biggl( {{{\biggl\vert {\frac{{\partial v}}{ {\partial{x_{i}}}}} \biggr\vert }^{{p_{i}} - 2}}\frac{{\partial v}}{ {\partial{x_{i}}}}} \biggr) = \sum _{i = 1}^{n} {{f_{2}}(x)} } {u^{{p_{i}}}},\quad x \in\Omega, $$

*must change sign*.

### Proof

Suppose that *v* to (2.7) does not change sign, without loss of generality, let $v > 0$ in Ω. By (2.6), (2.7), and (1.7), we observe

$$\begin{aligned} 0&\leq \int_{\Omega}{L(u,v)\,dx} = \int_{\Omega}{R(u,v)\,dx} \\ & = \sum_{i = 1}^{n} { \int_{\Omega}{{{\biggl\vert {\frac{{\partial u}}{ {\partial{x_{i}}}}} \biggr\vert }^{{p_{i}}}}} \,dx} - \sum_{i = 1}^{n} { \int _{\Omega}{\frac{\partial}{ {\partial{x_{i}}}} \biggl( {\frac{{{u^{{p_{i}}}}}}{ {{v^{{p_{i}} - 1}}}}} \biggr){{\biggl\vert {\frac{{\partial v}}{ {\partial{x_{i}}}}} \biggr\vert }^{{p_{i}} - 2}} \frac{{\partial v}}{ {\partial{x_{i}}}}} \,dx} \\ &= \sum_{i = 1}^{n} { \int_{\Omega}{{{\biggl\vert {\frac{{\partial u}}{ {\partial{x_{i}}}}} \biggr\vert }^{{p_{i}}}}} \,dx} + \sum_{i = 1}^{n} { \int _{\Omega}{\frac{{{u^{{p_{i}}}}}}{ {{v^{{p_{i}} - 1}}}}\frac{\partial}{ {\partial{x_{i}}}} \biggl( {{{ \biggl\vert {\frac{{\partial v}}{ {\partial{x_{i}}}}} \biggr\vert }^{{p_{i}} - 2}} \frac{{\partial v}}{ {\partial{x_{i}}}}} \biggr)\,dx} } \\ &= \sum_{i = 1}^{n} { \int_{\Omega}{ \bigl( {{f_{1}}(x) - {f_{2}}(x)} \bigr){u^{{p_{i}}}}} \,dx} \\ &< 0, \end{aligned}$$

which is a contradiction. This completes the proof. □

## Proof of Theorem 1.3

To prove Theorem 1.3, we need a lemma from Theorem 1.1.

### Lemma 3.1

*If there exist a constant* ${k_{i}} > 0$ *and a function* ${h_{i}}(x)$, $i = 1, \ldots,n$, *such that a differentiable function* $v>0$ *in the set* Ω *satisfies*

3.1 $$ - \frac{\partial}{ {\partial{x_{i}}}} \biggl( {{{\biggl\vert {\frac{{\partial v}}{ {\partial{x_{i}}}}} \biggr\vert }^{{p_{i}} - 2}}\frac{{\partial v}}{ {\partial{x_{i}}}}} \biggr) \geq{k_{i}} {h_{i}}(x){v^{{p_{i}} - 1}}, $$

*then*, *for any* $0 \leq u \in C_{0}^{1}(\Omega)$, *we have*

3.2 $$ \sum_{i = 1}^{n} { \int_{\Omega}{{{\biggl\vert {\frac{{\partial u}}{ {\partial{x_{i}}}}} \biggr\vert }^{{p_{i}}}}} \,dx} \geq\sum_{i = 1}^{n} {{k_{i}} \int_{\Omega}{{h_{i}}(x){u^{{p_{i}}}}} \,dx}. $$

### Proof

By (3.1) and (1.7), we see

$$\begin{aligned} 0&\leq \int_{\Omega}{L(u,v)\,dx} = \int_{\Omega}{R(u,v)\,dx} \\ & = \sum_{i = 1}^{n} { \int_{\Omega}{{{\biggl\vert {\frac{{\partial u}}{ {\partial{x_{i}}}}} \biggr\vert }^{{p_{i}}}}} \,dx} - \sum_{i = 1}^{n} { \int _{\Omega}{\frac{\partial}{ {\partial{x_{i}}}} \biggl( {\frac{{{u^{{p_{i}}}}}}{ {{v^{{p_{i}} - 1}}}}} \biggr){{\biggl\vert {\frac{{\partial v}}{ {\partial{x_{i}}}}} \biggr\vert }^{{p_{i}} - 2}} \frac{{\partial v}}{ {\partial{x_{i}}}}} \,dx} \\ &= \sum_{i = 1}^{n} { \int_{\Omega}{{{\biggl\vert {\frac{{\partial u}}{ {\partial{x_{i}}}}} \biggr\vert }^{{p_{i}}}}} \,dx} + \sum_{i = 1}^{n} { \int _{\Omega}{\frac{{{u^{{p_{i}}}}}}{ {{v^{{p_{i}} - 1}}}}\frac{\partial}{ {\partial{x_{i}}}} \biggl( {{{ \biggl\vert {\frac{{\partial v}}{ {\partial{x_{i}}}}} \biggr\vert }^{{p_{i}} - 2}} \frac{{\partial v}}{ {\partial{x_{i}}}}} \biggr)\,dx} } \\ &\leq\sum_{i = 1}^{n} { \int_{\Omega}{{{\biggl\vert {\frac{{\partial u}}{ {\partial{x_{i}}}}} \biggr\vert }^{{p_{i}}}}} \,dx} - \sum_{i = 1}^{n} {{k_{i}} \int _{\Omega}{{h_{i}}(x){u^{{p_{i}}}}} \,dx}, \end{aligned}$$

which implies (3.2). □

### Proof of Theorem 1.3

Without loss of generality, we let $0 \leq u \in C_{0}^{\infty}$. To use Lemma 3.1, we introduce the auxiliary function

3.3 $$ v = \prod_{j = 1}^{n} {{{ \vert {{x_{j}}} \vert }^{{\beta_{j}}}}} : = {\vert {{x_{i}}} \vert ^{{\beta_{i}}}} {\overline{v} _{i}}, $$

where ${\beta_{j}} = \frac{{{p_{j}} - 1}}{{{p_{j}}}}$ and ${\overline{v} _{i}} = \prod_{j = 1,j \ne i}^{n} {{{\vert {{x_{j}}} \vert }^{{\beta_{j}}}}} $, hence

$$\begin{aligned}& \frac{{\partial v}}{ {\partial{x_{i}}}} = {\beta_{i}} {\bar{v}_{i}} {\vert {{x_{i}}} \vert ^{{\beta_{i}} - 2}} {x_{i}}, \\& {\biggl\vert {\frac{{\partial v}}{ {\partial{x_{i}}}}} \biggr\vert ^{{p_{i}} - 2}}= { \beta_{i}}^{{p_{i}} - 2}{\bar{v}_{i}}^{{{p_{i}} - 2}}{\vert {{x_{i}}} \vert ^{{\beta_{i}}{p_{i}} - 2{\beta _{i}} - {p_{i}} + 2}}, \\& {\biggl\vert {\frac{{\partial v}}{ {\partial{x_{i}}}}} \biggr\vert ^{{p_{i}} - 2}} \frac{{\partial v}}{ {\partial{x_{i}}}} = {\beta_{i}}^{{p_{i}} - 1}{\overline{v} _{i}}^{{{p_{i}} - 1}}{\vert {{x_{i}}} \vert ^{{\beta_{i}}{p_{i}} - {\beta_{i}} - {p_{i}}}} {x_{i}}, \end{aligned}$$

and

3.4 $$ - \frac{\partial}{ {\partial{x_{i}}}} \biggl( {{{\biggl\vert {\frac{{\partial v}}{ {\partial{x_{i}}}}} \biggr\vert }^{{p_{i}} - 2}}\frac{{\partial v}}{ {\partial{x_{i}}}}} \biggr) = { \biggl( { \frac{{{p_{i}} - 1}}{ {{p_{i}}}}} \biggr)^{{p_{i}}}}\frac{{{v^{{p_{i}} - 1}}}}{ {{{\vert {{x_{i}}} \vert }^{{p_{i}}}}}}. $$

Taking ${k_{i}} = { ( {\frac{{{p_{i}} - 1}}{{{p_{i}}}}} )^{{p_{i}}}}$ and ${h_{i}}(x) = \frac{1}{{{{\vert {{x_{i}}} \vert }^{{p_{i}}}}}}$, and using Lemma 3.1, we obtain (1.8). □

### Corollary 3.2

*For* $u \in C_{0}^{1} ( A )$, *it follows that*

3.5 $$ \int_{A} {{{\vert {\nabla u} \vert }^{2}}} \,dx \geq\frac{{{n^{2}}}}{ 4} \int_{A } {\frac{{{{\vert u \vert }^{2}}}}{ {{{\vert x \vert }^{2}}}}} \,dx. $$

### Proof

Letting ${p_{i}} = 2$ ($i = 1, \ldots,n$) in (1.8) and noting the elementary inequality

3.6 $$ n{ \Biggl( {\sum_{i = 1}^{n} { \frac{1}{ {{a_{i}}}}} } \Biggr)^{ - 1}} \leq\frac{1}{ n} \Biggl( {\sum _{i = 1}^{n} {{a_{i}}} } \Biggr) \quad \mbox{for } {a_{i}} \geq0,i = 1, \ldots,n, $$

we have by taking ${a_{i}} = {\vert {{x_{i}}} \vert ^{2}}$,

$$\begin{aligned} \int_{A} {{{\vert {\nabla u} \vert }^{2}}} \,dx &= \sum_{i = 1}^{n} { \int _{A} {{{\biggl\vert {\frac{{\partial u}}{ {\partial{x_{i}}}}} \biggr\vert }^{2}}} \,dx} \\ & \geq\frac{1}{ 4} \int_{A} {{{\vert u \vert }^{2}} \Biggl( {\sum _{i = 1}^{n} {\frac{1}{ {{{\vert {{x_{i}}} \vert }^{2}}}}} } \Biggr)} \,dx \\ &\geq\frac{1}{ 4} \int_{A} {{{\vert u \vert }^{2}} \biggl( { \frac{{{n^{2}}}}{ {\sum_{i = 1}^{n} {{{\vert {{x_{i}}} \vert }^{2}}} }}} \biggr)} \,dx \\ &= \frac{{{n^{2}}}}{ 4} \int_{A} {\frac{{{{\vert u \vert }^{2}}}}{ {{{\vert x \vert }^{2}}}}} \,dx. \end{aligned}$$

 □

### Corollary 3.3

*If* $p > 2$, *then*, *for* $u \in C_{0}^{1} ( {A} )$, *it follows that*

3.7 $$ \int_{A} {{{\vert {\nabla u} \vert }^{p}}} \,dx \geq{ \biggl( {\frac{{p - 1}}{ p}} \biggr)^{p}} {n^{\frac{{p + 2}}{2}}} \int_{A} {\frac{{{{\vert u \vert }^{p}}}}{ {{{\vert x \vert }^{p}}}}} \,dx. $$

### Proof

Let ${p_{i}} = p > 2$ ($i = 1, \ldots,n$) in (1.8). Recall the inequality

$$\sum_{i = 1}^{n} {{a_{i}}^{2}} \leq{ \Biggl( {\sum_{i = 1}^{n} {{a_{i}}^{p}} } \Biggr)^{\frac{2}{p}}} {n^{\frac{{p - 2}}{p}}} \quad \mbox{for } {a_{i}} \geq0,i = 1, \ldots,n, $$

which gives

3.8 $$ \sum_{i = 1}^{n} {{a_{i}}^{p}} \geq{n^{ - \frac{{p - 2}}{2}}} { \Biggl( {\sum _{i = 1}^{n} {{a_{i}}^{2}} } \Biggr)^{\frac{p}{2}}}. $$

Taking ${a_{i}} = \frac{1}{{\vert {{x_{i}}} \vert }}$ in (3.8), it implies by (3.6) that

3.9 $$ \sum_{i = 1}^{n} { \frac{1}{ {{{\vert {{x_{i}}} \vert }^{p}}}}} \geq{n^{ - \frac{{p - 2}}{2}}} { \Biggl( {\sum _{i = 1}^{n} {\frac{1}{ {{{\vert {{x_{i}}} \vert }^{2}}}}} } \Biggr)^{\frac{p}{2}}} \geq{n^{ - \frac{{p - 2}}{2}}} { \biggl( {\frac{{{n^{2}}}}{ {\sum_{i = 1}^{n} {{{\vert {{x_{i}}} \vert }^{2}}} }}} \biggr)^{\frac{p}{2}}} = {n^{\frac{{p + 2}}{2}}}\frac{1}{ {{{\vert x \vert }^{p}}}}. $$

Putting (3.9) into the right-hand side of (1.8),

3.10 $$ \sum_{i = 1}^{n} {{{ \biggl( { \frac{{p - 1}}{ p}} \biggr)}^{p}} \int_{A} {\frac{{{{\vert u \vert }^{p}}}}{ {{{\vert {{x_{i}}} \vert }^{p}}}}} \,dx} \geq{ \biggl( { \frac{{p - 1}}{ p}} \biggr)^{p}} {n^{\frac{{p + 2}}{2}}} \int_{A} {\frac{{{{\vert u \vert }^{p}}}}{ {{{\vert x \vert }^{p}}}}} \,dx. $$

On the other hand,

3.11 $$ \int_{A} {\sum_{i = 1}^{n} {{{\biggl\vert {\frac{{\partial u}}{ {\partial{x_{i}}}}} \biggr\vert }^{p}}} } \,dx \leq \int_{A } {{{ \Biggl( {\sum_{i = 1}^{n} {{{\biggl\vert {\frac{{\partial u}}{ {\partial{x_{i}}}}} \biggr\vert }^{2}}} } \Biggr)}^{\frac{p}{2}}}} \,dx = \int_{A } {{{\vert {\nabla u} \vert }^{p}}} \,dx. $$

Hence (3.7) is proved via (3.10) and (3.11). □
